# Supplementary material for: Targeting macrophage Histone deacetylase 3 stabilizes atherosclerotic lesions
Source: EMBO Mol Med. 2014 Jul 9;6(9):1124–32. doi: 10.15252/emmm.201404170 (PMC4197860; doi:10.15252/emmm.201404170)
Supplement: Supplementary file 1 — Supplementary Figure S1 [file emmm0006-1124-SD1.pdf]

Figure S1

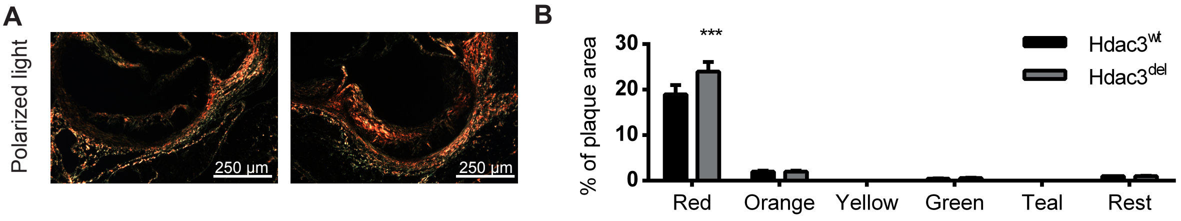

Figure S1. Myeloid Hdac3 deletion results in more stable collagen in atherosclerotic lesions.

A and B. Using polarization microscopy on Sirius Red-stained lesions, collagen subtypes were quantified (n=19/18). Statistical analysis was performed using 2-way ANOVA (P = 0.0002). Error bars indicate SEM.
