# Supplementary material for: Targeting macrophage Histone deacetylase 3 stabilizes atherosclerotic lesions
Source: EMBO Mol Med. 2014 Jul 9;6(9):1124–32. doi: 10.15252/emmm.201404170 (PMC4197860; doi:10.15252/emmm.201404170)
Supplement: Supplementary file 2 — Supplementary Figure S2 [file emmm0006-1124-SD2.pdf]

Figure S2

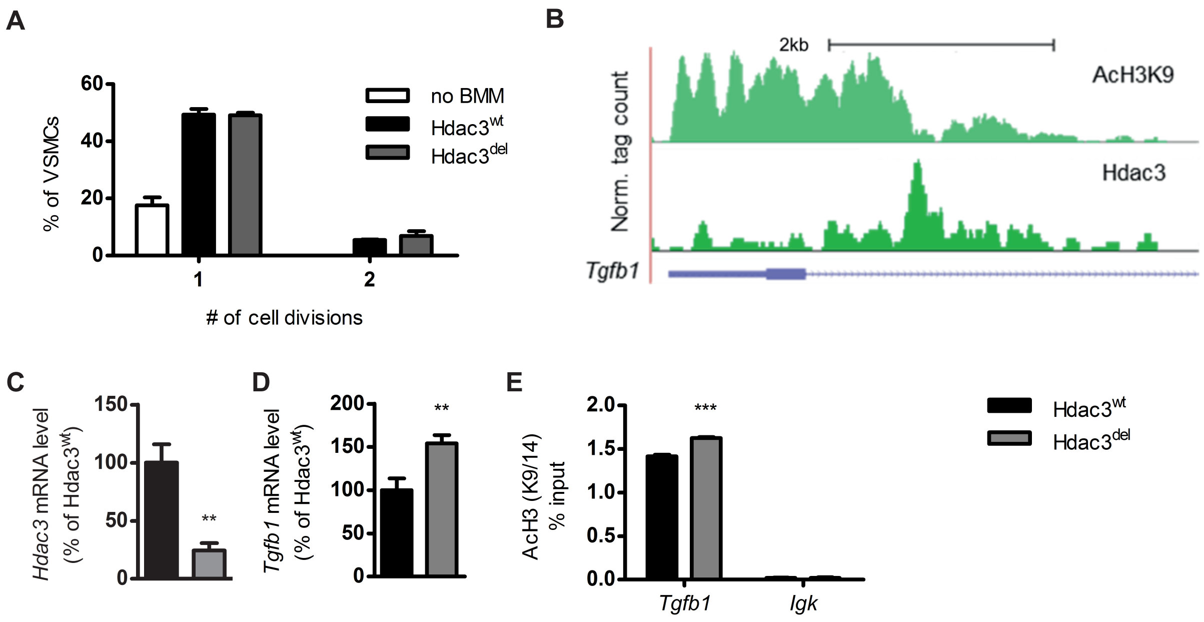

Figure S2. VSMC proliferation, Hdac3 enrichment at the *Tgfb1* promoter and histone acetylation in peritoneal macrophages on the *Tgfb1* promoter.

A. Cell proliferation of VSMCs after 24 hour incubation with oxLDL-stimulated BMM supernatants was determined with CFSE on FACS, the number of cell divisions is presented here.

B. UCSC genome browser image showing normalized tag counts for H3K9Ac and Hdac3 ChIP-seq at the 5'-end of the *Tgfb1* locus.

C-E. PMs from HCD fed Hdac3^{wt} and Hdac3^{del}-transplanted LDLR$^{-/-}$ mice (n=4/5) were harvested.

C. *Hdac3* gene expression (P = 0.0058) in peritoneal macrophages was measured and normalized to household genes.

D. *Tgfb1* gene expression (P = 0.0022) in peritoneal macrophages was measured and normalized to household genes.

E. ChIP for AcH3K9/14 was performed on peritoneal macrophages (P = 0.0005, 2-way ANOVA).

Unpaired t-test and two-way ANOVA with Bonferroni correction was performed for statistical analysis. Error bars indicate SEM.
