# Supplementary material for: Targeting macrophage Histone deacetylase 3 stabilizes atherosclerotic lesions
Source: EMBO Mol Med. 2014 Jul 9;6(9):1124–32. doi: 10.15252/emmm.201404170 (PMC4197860; doi:10.15252/emmm.201404170)
Supplement: Supplementary file 3 — Supplementary Figure S3 [file emmm0006-1124-SD3.pdf]

Figure S3

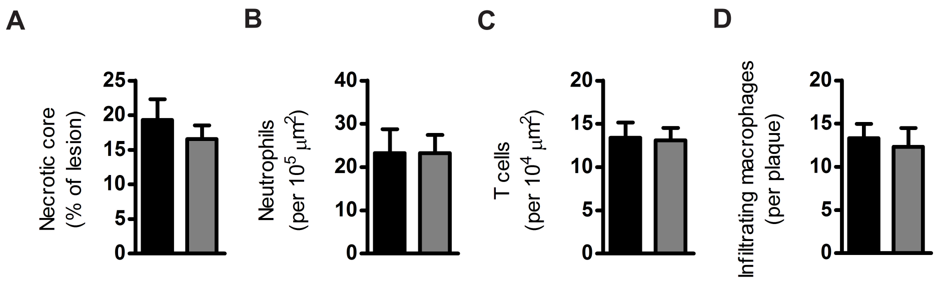

Figure S3. Myeloid Hdac3 deletion does not affect inflammatory cell infiltration in the atherosclerotic lesions

A. Necrotic core was calculated as a percentage of lesion area (n=10/10).

B. Neutrophils (NIMP1$^+$ cells, n=19/18) were counted in the atherosclerotic lesions.

C. T cells (CD3$^+$ cells, n=19/18) were counted in the atherosclerotic lesions.

D. Infiltrating macrophages (ERMP58$^+$ cells, n=19/18) were counted in the atherosclerotic lesions. Unpaired t-test was performed for statistical analysis. Error bars indicate SEM.
