# Supplementary material for: Targeting macrophage Histone deacetylase 3 stabilizes atherosclerotic lesions
Source: EMBO Mol Med. 2014 Jul 9;6(9):1124–32. doi: 10.15252/emmm.201404170 (PMC4197860; doi:10.15252/emmm.201404170)
Supplement: Supplementary file 4 — Supplementary Figure S4 [file emmm0006-1124-SD4.pdf]

Figure S4

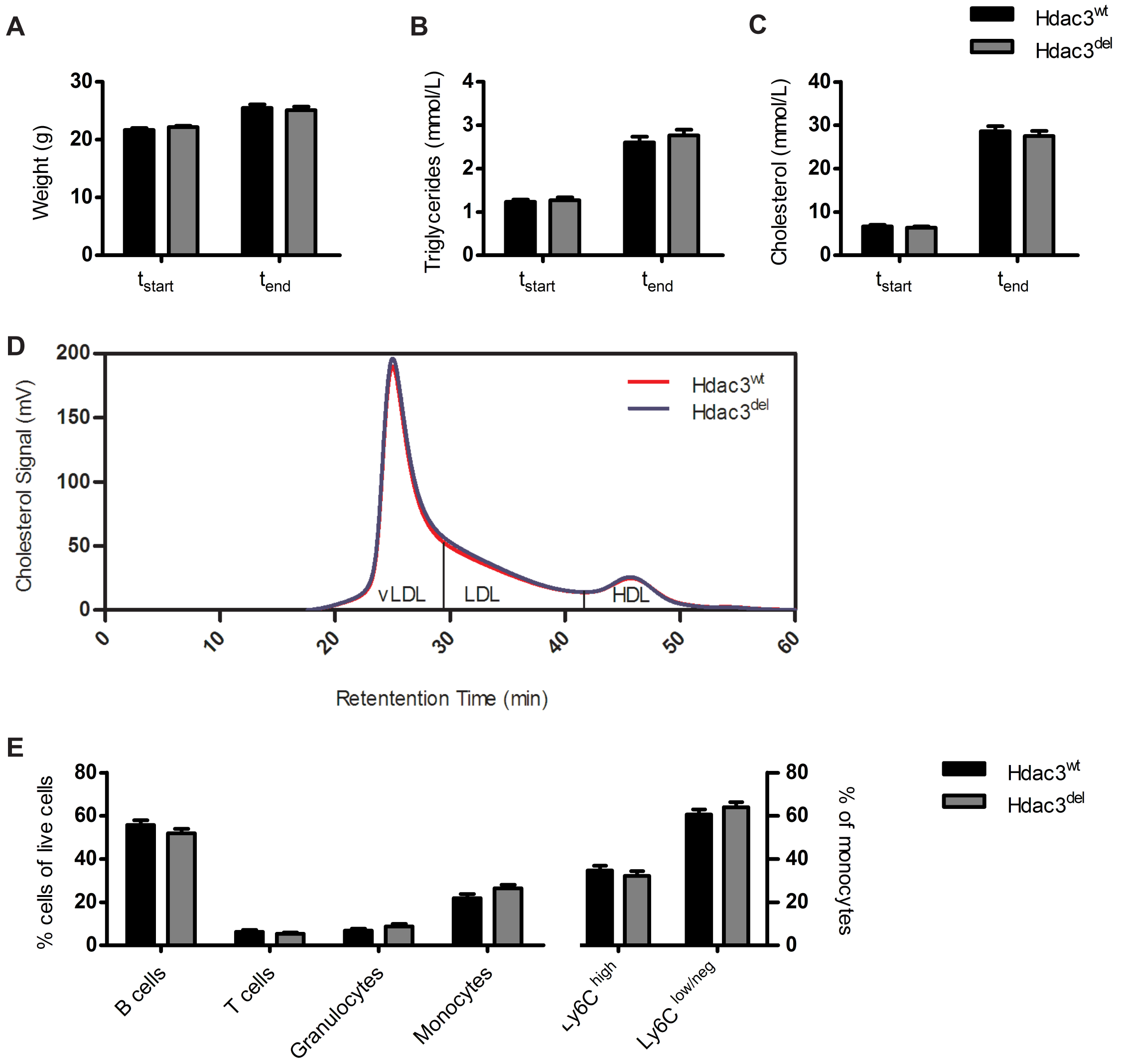

Figure S4. Weight, lipid levels and immune cell counts are similar in transplanted LDLR$^{-/-}$ mice.

A. Weight was measured before the start of the HCD and when the mice were sacrificed.

B. Triglycerides levels were determined in the plasma.

C. Cholesterol levels were determined in the plasma.

D. Cholesterol subfractions were determined in the plasma.

E. Leukocyte proportions in the blood were evaluated when the mice were sacrificed.
