# Supplementary material for: Targeting macrophage Histone deacetylase 3 stabilizes atherosclerotic lesions
Source: EMBO Mol Med. 2014 Jul 9;6(9):1124–32. doi: 10.15252/emmm.201404170 (PMC4197860; doi:10.15252/emmm.201404170)
Supplement: Supplementary file 5 — Supplementary Figure S5 [file emmm0006-1124-SD5.pdf]

Figure S5

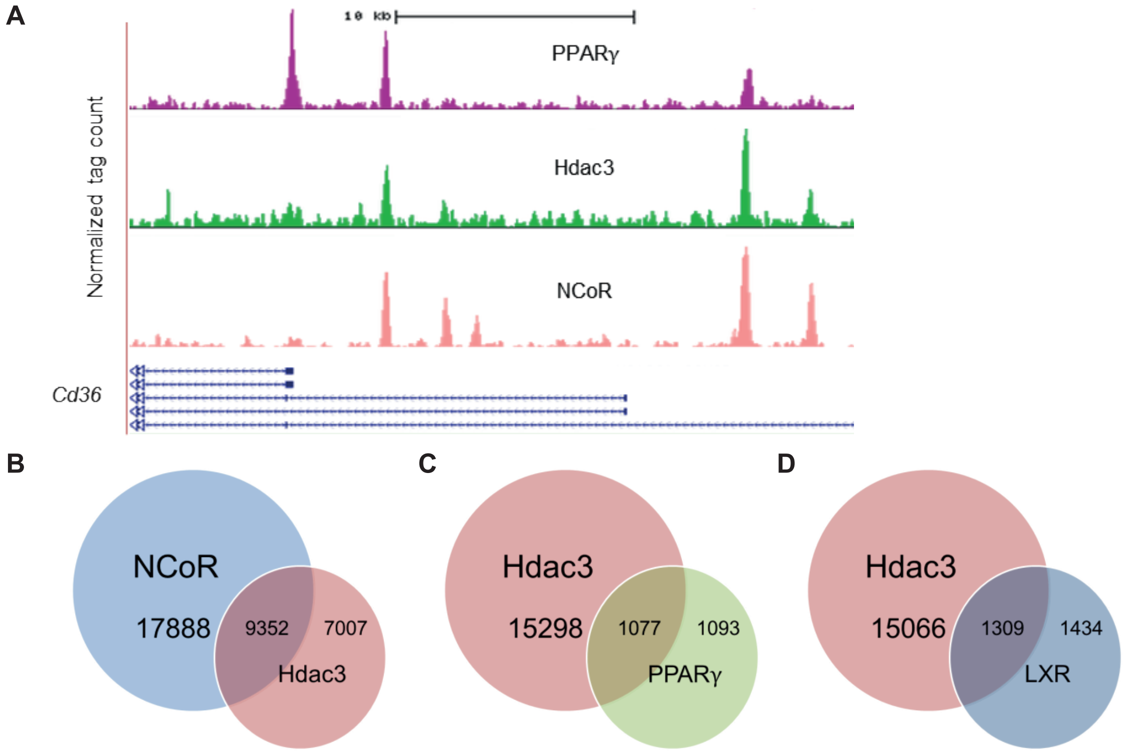

Figure S5. Hdac3 peaks overlap with PPARγ and LXR peaks

A. UCSC genome browser image illustrating normalized tag counts for PPARγ (Lefterova et al., 2010), Hdac3 (Mullican et al., 2011) and NCoR (Barish et al., 2012) ChIP-seq at the *Cd36* locus as an example of a PPARγ target gene where PPARγ, Hdac3 and NCoR bind to the same locus.

B-D. Venn diagrams representing the overlap between the NCoR and Hdac3 peaks (B), the Hdac3 and PPARγ peaks (C) and the Hdac3 and LXR (Li et al., 2013) peaks (D).
