# Supplementary material for: Targeting macrophage Histone deacetylase 3 stabilizes atherosclerotic lesions
Source: EMBO Mol Med. 2014 Jul 9;6(9):1124–32. doi: 10.15252/emmm.201404170 (PMC4197860; doi:10.15252/emmm.201404170)
Supplement: Supplementary file 6 — Supplementary Figure S6 [file emmm0006-1124-SD6.pdf]

Figure S6

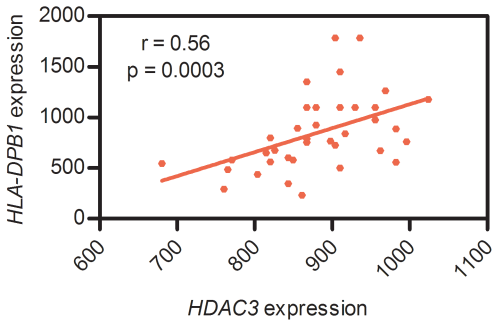

Figure S6. *HDAC3* correlates with *HLA-DPB1* in human atherosclerotic lesions. Correlation of *HDAC3* with pro-inflammatory macrophage marker *HLA-DPB1* in gene expression data from human atherosclerotic lesions (n=40). Statistical analysis was performed with Pearson's correlation test.
